# Dental Visiting Patterns and Their Associations With Dental Caries and Periodontal Diseases

**DOI:** 10.1002/cre2.70087

**Published:** 2025-02-12

**Authors:** Najith Amarasena, Liana Luzzi, Sergio Chrisopoulos, Gloria Mejia

**Affiliations:** ^1^ Australian Research Centre for Population Oral Health, Adelaide Dental School, Faculty of Health and Medical Sciences The University of Adelaide Adelaide South Australia Australia

**Keywords:** dental caries, dental visiting patterns, periodontal diseases

## Abstract

**Objectives:**

This study was conducted to describe the dental visiting patterns and ascertain their associations with clinically assessed dental caries and periodontal disease levels of dentate Australian adults.

**Materials and Methods:**

A three‐stage stratified probability sampling design was used to randomly select a cross‐section of Australians aged 15+ years. Self‐reported oral health and related information was obtained using questionnaire‐based interviews. Dental visiting patterns studied were usual frequency of dental visits, usual reason for dental visit, and use of a regular dentist. Oral examinations were conducted following a standardized protocol in public dental clinics. A total of 5022 dentate adults aged 15+ years who were interviewed and orally examined were included in the current analysis.

**Results:**

Higher proportions of adults with lower education levels and no dental insurance usually visited for a dental problem, made dental visits less frequently, and did not use a regular dentist. Individuals who were usually visiting for a dental problem, making dental visits less frequently, and not using a regular dentist had higher levels of dental caries and periodontal diseases.

**Conclusions:**

Dental visiting patterns of Australian adults were associated with their socioeconomic backgrounds. Usually visiting for a dental problem, making dental visits less frequently, and not using a regular dentist were more likely to be associated with higher levels of dental caries and periodontal diseases. These findings may help clinicians to recognize patients at increased risk for dental caries and periodontal diseases while apprising policy makers to plan and implement programs for dental service provision.

## 1 Introduction

Dental caries, periodontal diseases, and oral cancers are the main oral diseases and the most ubiquitously occurring diseases among over 300 diseases and conditions affecting humankind (World Health Organization 2022; Peres et al. 2019). Global oral disease burden has been on the rise throughout the past 3–4 decades and almost 3.5 billion people have been afflicted with oral diseases worldwide (World Health Organization 2022; Peres et al. 2019). Among oral diseases, dental caries is the most prevalent disease, with about 2.8 billion cases of untreated dental decay, which is followed by severe periodontitis, with nearly one billion cases (World Health Organization 2022). The situation in Australia is no different, with the latest reports indicating that the prevalence of untreated dental caries of permanent teeth among Australian adults and children was 32% (Australian Research Centre for Population Oral Health (ARCPOH) 2019) and 27% (Ha et al. 2012), respectively, with 11% of children having untreated decay in their primary teeth (Ha et al. 2012) whereas the prevalence of gingivitis and periodontitis among Australian adults was 29% and 30%, respectively (ARCPOH 2019).

Dental visiting patterns, including the frequency of dental visits, usual reason for dental visits, and use of a regular dentist, generally reflect access to dental care (ARCPOH 2019; Brennan et al. 2020). Dental visiting frequency or rather the most recent dental visit can point to the recency of the last visit and current oral healthcare‐seeking behavior, whereas long‐term dental visiting patterns are reflected by usual reason for dental visits made either for a regular check‐up or a dental problem needing treatment (ARCPOH 2019; Brennan et al. 2020). A pattern of visiting the same dental practitioner/practice, on the other hand, indicates a perseverance in obtaining dental care while maintaining a persistent rapport with a given practitioner/practice (ARCPOH 2019; Brennan et al. 2020). This shares essentially the same concept with the “dental home,” which is widely cited in the literature and defined as the ongoing relationship between the dentist and the patient inclusive of every aspect of oral health (Girish Babu and Doddamani 2012). These visiting patterns can provide an opportunity for prevention, early diagnosis, and prompt treatment of oral diseases before they progress (ARCPOH 2019; Brennan et al. 2020; Riley et al. 2013). Visiting a dentist more frequently has been associated with reduced tooth loss as well as improved oral functional ability (Sheiham 1977) and inversely associated with worsening self‐reported oral health over 2 years (Brennan et al. 2012). On the other hand, it has been shown that patients with non‐regular dental visiting and long‐term routine dental patterns, respectively, have lower self‐rated oral health (Crocombe et al. 2011) and improved oral health‐related quality of life (Thomson et al. 2010). Nonetheless, dental visiting patterns can also be affected, mainly by financial barriers, including inability to afford the cost of dental treatment, and nonfinancial barriers such as oral health literacy, dental anxiety, and accessibility to and availability of oral health services (Riley et al. 2013; Locker et al. 2011; Gupta and Vujicic 2019).

The Australian dental healthcare system is dominated by a fee‐for‐service private practice system and consisted of a dental workforce that includes nearly three‐quarters of dentists, followed by oral health therapists, dental hygienists, dental prosthetists, and dental therapists. The dental public sector, with school dental service and public dental care for adults being its two main components, contributes to less than 20% of dental healthcare provision in Australia. School dental services provide dental care to about 50% of primary school‐aged children, whereas the coverage is lower among secondary school‐aged children. Public dental care for adults, available only to means‐tested eligible adults holding a government concession card, is provided through dental hospitals, community health centers, and regional facilities. In Australia, use of dental services at the national level was reported recently and Australian adults from low socioeconomic backgrounds, who resided in remote locations and did not have dental insurance, were at a disadvantage with regard to access to dental care. A lower proportion of them visited a dentist in the last 12 months, usually visited for a check‐up, and used a regular dentist (ARCPOH 2019; Brennan et al. 2020). Nonetheless, associations of dental visiting patterns of dentate Australian adults with their clinically recorded dental disease levels have not been studied at the national level. Accordingly, the present study was conducted with the objectives of (1) describing the dental visiting patterns, including usual frequency of dental visits, usual reason for dental visit, and use of a regular dentist, of dentate adults in Australia and (2) ascertaining the associations of such visiting patterns with clinically assessed dental caries and periodontal disease levels of the same population, based on the data from the National Study of Adult Oral Health (NSAOH) 2017–18.

## 2 Materials and Methods

A detailed description of the study methodology, including computation of sample size, has been reported elsewhere (ARCPOH 2019; Chrisopoulos et al. 2020; Amarasena et al. 2021). The total sample size targeted for interviews to comply with 7200 oral examinations was 15,200. In brief, a three‐stage stratified probability sampling design was used to conduct a cross‐sectional study of a random sample of Australians aged 15 years and above from all Australian states and territories. Sampling of postcodes within states/territories, mostly by means of systematic sampling with probability of selection proportional to the number of households within the postcode, was followed by selecting individuals aged 15 years and above within selected postcodes from the Medicare database by Services Australia, formerly known as the Australian Government Department of Human Services (DHS). Trained interviewers, using a questionnaire based on previous surveys (Chrisopoulos et al. 2020; Amarasena et al. 2021), conducted interviews online or by telephone (CATI—computer‐assisted telephone interview). Interviewed subjects provided verbal consent before answering questions. Parental/guardian consent was obtained for participants aged 15–17 years. All examined subjects provided signed, informed consent before the examination (parents/guardians of participants aged 15–17 years provided signed, informed consent before the examination).

Information on age, sex, Indigenous identity, residential location, schooling/educational qualifications, eligibility for public dental care, dental insurance, and dental visiting patterns was obtained through interviews. Age was categorized into four groups (15–34, 35–54, 55–74, and 75+ years) based on four “dental generations of Australians” who have had different historical influences on their oral health. The earliest generation (75+ year olds) reached adulthood during a period where oral disease was widespread and extraction of teeth was the most common mode of treatment. Australians aged 55–74 and 35–54 years, each born in two‐decade periods after the earliest generation, were more likely to retain teeth, although they experienced historically high rates of dental caries. The youngest generation of Australians aged 15–34 years has had exposure to more oral health promotion and prevention than any preceding generation, in particular through fluoridated toothpastes and drinking water. Dental visiting patterns included usual frequency of dental visits (≥ 1/year; < 1/year), usual reason for dental visit (check‐up; problem), and use of a regular dentist/practice (yes; no). A detailed description of the questions relevant to dental visiting patterns is included in a Supporting Information. Usual frequency of dental visits reflects dental healthcare‐seeking behavior, whereas long‐term patterns of dental visiting are denoted by the usual reason for visit. Continuity of dental care and an ongoing relationship with a particular dentist are reflected by a pattern of usually attending the same dentist/practice.

Forty dental practitioners working in the state/territory public dental services conducted oral examinations. A 2‐day training and calibration session, which was conducted by the dental staff from the University of Adelaide, was undertaken by all 40 examiners, in addition to the separate training sessions held for the examination teams from each state and territory. A manual and a DVD detailing the study protocol, including criteria and coding for the examination, were provided to each examiner before the scheduled training session. Twenty five examiners were involved in the assessment for inter‐examiner reliability by testing against the gold‐standard examiners who were the study trainers. Accordingly, replicate pairs of examinations were conducted with 101 study participants that yielded intra‐class correlation coefficients ranging from 0.75 to 0.96 for diagnosing periodontal diseases and dental caries. Dentate participants who consented to an examination were included for oral examinations. Oral examinations were conducted in standard dental chairs with illumination provided by the overhead dental light of the chair. Additionally, examiners used an intra‐oral mirror with its own battery‐powered light source that facilitated recording of enamel cavities with visual examination. The participants were not specifically asked to brush their teeth before oral examination. Dental caries and periodontal diseases were assessed as follows:

### 2.1 Dental Caries (Coronal Caries)

Dental caries was assessed on five tooth surfaces (mesial, buccal, distal, lingual, and occlusal/incisal) using visual criteria without an explorer. In the present study, the prevalence of dental caries experience, the severity of untreated dental caries (the burden of disease), and the severity of dental caries experience (lifetime experience of dental caries), respectively, were indicated by the proportion of adults with untreated coronal decay, the number of decayed tooth surfaces (DS) per person, and the number of decayed, missing, or filled tooth surfaces (DMFS) per person. Once caries has reached dentine, through enamel, the resulting cavity cannot heal, and treatment of dental decay, either as a filling or extraction, leaves a permanent sign of disease and, therefore, DMFS implies lifetime experience of dental caries (Chrisopoulos et al. 2020).

### 2.2 Periodontal Diseases

Participants who had no medical contraindications for periodontal probing were clinically assessed for periodontal diseases. A medical history questionnaire was used to exclude adults with certain medical conditions (persons who need prophylactic antibiotic cover before dental check‐up/care, persons with heart diseases, rheumatic fever, kidney disease, hemophilia, pacemaker/defibrillator, transplanted organs, and hip bone/joint replacement in the past 3 months) from periodontal examination. The following criteria were used to assess gingivitis and periodontitis, the two types of periodontal disease.

#### 2.2.1 Gingivitis

The gingival index of Löe and Silness (Löe and Silness 1963) was used to assess inflammation of the marginal gingival tissues around six index teeth (if present: the most anterior molar tooth in each dental quadrant + right upper central incisor + left lower central incisor).

#### 2.2.2 Periodontitis

Periodontal tissue destruction was assessed using the US National Health and Nutrition Examination Survey (NHANES) methods (NIDCR 2002). A periodontal probe was used to assess three aspects (mesio‐buccal, mid‐buccal, and disto‐buccal) of all teeth present, except third molars (wisdom teeth). A case definition developed by the US Centers for Disease Control and Prevention (CDC) and the American Academy of Periodontology (AAP) was used to describe the prevalence of moderate and severe periodontitis (NIDCR 2002). Accordingly, moderate periodontitis was defined as the presence of either at least two proximal sites, not on the same tooth, with attachment loss of 4 mm or more, or at least two such sites that had pockets of 5 mm or more. Having at least two proximal sites not on the same tooth with attachment loss of 6 mm or more, plus at least one periodontal pocket with a depth of 5 mm or more was defined as severe periodontitis.

All data were weighted to population benchmarks to ensure representativeness of the target population (Chrisopoulos et al. 2020). Data files were managed and summary variables were computed using SAS software version 9.4 (SAS 9.4; SAS Institute Inc. Cary, NC, USA). The estimates, including proportions and means, were calculated, whereas their reliability was expressed via 95% confidence intervals (95% CI).

## 3 Results

A total of 5022 dentate adults aged 15 years and over who were interviewed and orally examined were included for the current analysis. The number of persons who completed the interviews was 15,731 and the number of persons who were eligible for interviews was 39,651; therefore, the overall participation rate for interviews was 39.7% (15,731/39,651). The overall participation rate for oral examinations was 33.6%, which was calculated by dividing the number of persons examined (5022) by the number of interviewed persons who were dentate (14,944). Table 1 shows the dental visiting patterns, including the usual frequency of visits, usual reason for visit, and use of a regular dentist, of dentate Australian adults by their age and other explanatory variables. The usual frequency of dental visits was age‐related, with the proportion of individuals who usually make one or more dental visits per year increasing gradually across the age groups, from the lowest in 15–34‐year‐olds (48.2%) to the highest in ≥ 75‐year‐olds (63.0%). Conversely, the proportion of ≥ 75‐year‐olds who usually visit a dentist less than once a year was the lowest (37.0%) and that of 15–34‐year‐olds was the highest (51.8%). Residents in major cities, individuals with a higher year level of schooling as well as a degree or higher education qualification, and adults who had dental insurance usually make dental visits more frequently than their corresponding counterparts. Among all participants, the proportion of individuals who usually make one or more dental visits per year was the highest in dentally insured individuals (67.1%), followed by oldest adults aged ≥ 75 years (63.0%), whereas usually making less frequent dental visits was most common in dentally uninsured adults, followed by adults who had year 10 or less level of schooling, with 60.5% and 58.1% of them, respectively, usually visiting a dentist less than once a year.

**Table 1:** Dental visiting patterns of dentate Australian adults by age and other explanatory variables.

	*n*	% (95% CI)	Dental visiting patterns					
			Frequency of dental visits		Usual reason for visit		Use of a regular dentist	
			≥ 1/year % (95% CI)	< 1/year % (95% CI)	Check‐up % (95% CI)	Problem % (95% CI)	Yes % (95% CI)	No % (95% CI)
Age (years)								
15–34	1196	35.9 (33.2, 38.6)	48.2 (43.5, 52.9)	51.8 (47.1, 56.5)	69.2 (65.1, 73.0)	30.8 (27.0, 34.9)	63.3 (58.4, 68.0)	36.7 (32.0, 41.6)
35–54	1579	33.5 (31.1, 36.1)	50.4 (45.6, 55.1)	49.6 (44.9, 54.4)	59.2 (54.5, 63.8)	40.8 (36.2, 45.5)	74.8 (70.2, 78.8)	25.2 (21.2, 29.8)
55–74	1704	23.7 (22.0, 25.5)	56.8 (52.8, 60.7)	43.2 (39.3, 47.2)	53.7 (49.6, 57.8)	46.3 (42.2, 50.4)	82.9 (79.5, 85.9)	17.1 (14.1, 20.5)
≥ 75	428	6.9 (5.9, 8.0)	63.0 (54.2, 71.0)	37.0 (29.0, 45.8)	60.0 (52.0, 67.5)	40.0 (32.5, 48.0)	87.2 (78.8, 92.7)	12.8 (7.3, 21.2)
Sex								
Male	2190	49.8 (47.2, 52.4)	48.2 (44.1, 52.3)	51.8 (47.7, 55.9)	61.8 (58.1, 65.4)	38.2 (34.6, 41.9)	70.9 (66.9, 74.6)	29.1 (25.4, 33.1)
Female	2717	50.2 (47.6, 52.8)	55.7 (52.1, 59.2)	44.3 (40.8, 47.9)	61.2 (57.7, 64.5)	38.8 (35.5, 42.3)	76.1 (73.0, 79.0)	23.9 (21.0, 27.0)
Indigenous identity								
Nonindigenous	4826	98.4 (97.4, 99.0)	51.9 (49.1, 54.6)	48.1 (45.4, 50.9)	61.4 (58.7, 64.0)	38.6 (36.0, 41.3)	73.5 (70.8, 76.0)	26.5 (24.0, 29.2)
Indigenous	80	1.6 (1.0, 2.6)	62.4 (41.8, 79.4)	37.6 (20.6, 58.2)	65.8 (46.6, 80.8)	34.2 (19.2, 53.4)	79.6 (58.8, 91.4)	20.4 (8.6, 41.2)
Residential location								
Major cities	3183	67.3 (65.8, 68.8)	56.8 (53.3, 60.2)	43.2 (39.8, 46.7)	64.8 (61.5, 68.0)	35.2 (32.0, 38.5)	72.7 (69.3, 75.9)	27.3 (24.1, 30.7)
Rural/remote	1724	32.7 (31.2, 34.2)	42.1 (37.7, 46.6)	57.9 (53.4, 62.3)	54.6 (50.1, 59.0)	45.4 (41.0, 49.9)	75.7 (71.6, 79.3)	24.3 (20.7, 28.4)
Year level of schooling								
Year 10 or less	1157	25.6 (23.4, 27.8)	41.9 (37.5, 46.4)	58.1 (53.6, 62.5)	47.7 (43.0, 52.5)	52.3 (47.5, 57.0)	79.6 (75.4, 83.3)	20.4 (16.7, 24.6)
Year 11 or more	3715	74.4 (72.2, 76.6)	55.5 (52.2, 58.7)	44.5 (41.3, 47.8)	66.3 (63.3, 69.1)	33.7 (30.9, 36.7)	71.8 (68.6, 74.9)	28.2 (25.1, 31.4)
Highest qualification attained								
Degree or higher	1993	29.6 (27.1, 32.2)	60.8 (56.7, 64.7)	39.2 (35.3, 43.3)	74.7 (71.1, 77.9)	25.3 (22.1, 28.9)	71.2 (66.5, 75.4)	28.8 (24.6, 33.5)
Other/None	2857	70.4 (67.8, 72.9)	48.0 (44.8, 51.3)	52.0 (48.7, 55.2)	56.5 (53.2, 59.8)	43.5 (40.2, 46.8)	75.1 (71.9, 78.0)	24.9 (22.0, 28.1)
Eligibility for public dental care								
Eligible	1585	30.3 (28.0, 32.8)	48.4 (44.2, 52.7)	51.6 (47.3, 55.8)	49.4 (45.1, 53.7)	50.6 (46.3, 54.9)	73.7 (68.8, 78.1)	26.3 (21.9, 31.2)
Ineligible	3307	69.7 (67.2, 72.0)	53.3 (50.0, 56.6)	46.7 (43.4, 50.0)	66.6 (63.5, 69.6)	33.4 (30.4, 36.5)	73.5 (70.5, 76.3)	26.5 (23.7, 29.5)
Dental insurance								
Insured	2524	46.1 (43.3, 48.9)	67.1 (63.2, 70.7)	32.9 (29.3, 36.8)	75.3 (71.6, 78.6)	24.7 (21.4, 28.4)	83.5 (80.1, 86.4)	16.5 (13.6, 19.9)
Uninsured	2301	53.9 (51.1, 56.7)	39.5 (35.8, 43.3)	60.5 (56.7, 64.2)	49.3 (45.7, 52.8)	50.7 (47.2, 54.3)	66.0 (62.0, 69.7)	34.0 (30.3, 38.0)

Abbreviations: 95% CI = 95% confidence intervals, *n* = unweighted sample size.

With regard to usual reason for dental visit, the proportion of adults usually visiting either for a check‐up or problem varied across age groups. The proportion who visited for a check‐up was highest among 15–34‐year‐olds (69.2%) and lowest among 55–74‐year‐olds (53.7%). Higher proportions of adults who usually visit for a check‐up were seen among residents of major cities, participants with higher year level of schooling as well as educational qualifications, who were ineligible for public dental care, and who had dental insurance. Among all adults, the proportion who usually visit for a check‐up was highest among dentally insured individuals (75.3%), followed by persons who had a degree or higher level of educational qualification (74.7%), whereas the proportion who usually visit for a problem was highest among people who had year 10 or less level of education (52.3%), closely followed by dentally uninsured individuals (50.7%) and adults who were eligible for public dental care (50.6%). Dentally uninsured adults were almost 2 times more likely to be problem‐oriented visitors than adults who were dentally insured.

As for the use of a regular dentist, there was a gradient across age groups, with the lowest proportion of adults using a regular dentist being 15–34‐year‐olds (63.3%), which increased gradually across the age groups, with ≥ 75‐year‐olds being the highest proportion (87.2%) in that category. In addition, a higher proportion of dentally insured adults (83.5%) and adults who had year 10 or less level of schooling (79.6%) were using a regular dentist than their corresponding counterparts. Among all participants, the proportion who were not using a regular dentist was highest in young adults aged 15–34 years (36.7%), followed by adults without dental insurance (34.0%). Adults without dental insurance were nearly 2 times more likely to be not using a regular dentist than adults who had dental insurance.

### 3.1 Dental Caries and Dental Visiting Patterns

Table 2 depicts the proportion of dentate Australian adults with untreated coronal caries by age group and dental visiting patterns. With regard to the frequency of dental visits, the overall prevalence of untreated coronal caries was 22.1% and 24.4%, respectively, among the participants who usually made a dental visit once a year or more and less than once a year. Nonetheless, any obvious variation in untreated coronal caries experience by frequency of making dental visits was not evident in general or across age groups.

**Table 2:** Proportion of dentate Australian adults with untreated coronal caries by age group and dental visiting patterns.

			Age (years)				
	*n*	% (95% CI)	Total % (95% CI)	15–34% (95% CI)	35–54% (95% CI)	55–74% (95% CI)	≥ 75% (95% CI)
Frequency of dental visits (%)							
≥ 1/year	2653	52.0 (49.2, 54.7)	22.1 (17.9, 27.0)	20.7 (12.5, 32.3)	21.0 (14.5, 29.5)	25.1 (18.4, 33.2)	21.8 (13.1, 34.1)
< 1/year	2254	48.0 (45.3, 50.8)	24.4 (20.9, 28.2)	21.7 (16.4, 28.1)	27.8 (20.9, 36.0)	26.0 (18.2, 35.5)	16.9 (9.9, 27.2)
Usual reason for visit (%)							
Check‐up	3135	61.5 (58.8, 64.1)	24.3 (21.4, 27.5)	24.2 (19.3, 29.9)	25.4 (20.3, 31.3)	24.4 (19.6, 30.0)	19.5 (13.1, 28.0)
Problem	1796	38.5 (35.9, 41.2)	43.5 (39.3, 47.9)	43.7 (35.6, 52.2)	49.2 (42.5, 56.0)	39.4 (32.5, 46.7)	30.9 (20.1, 44.3)
Use of a regular dentist (%)							
Yes	3444	73.6 (71.0, 76.1)	27.1 (24.3, 30.1)	24.6 (19.6, 30.4)	30.2 (25.2, 35.7)	27.7 (23.1, 32.8)	22.1 (16.2, 29.4)
No	967	26.4 (23.9, 29.0)	34.6 (29.4, 40.1)	31.9 (23.6, 41.4)	40.1 (30.4, 50.5)	32.6 (24.1, 42.4)	30.6 (13.3, 55.8)

Abbreviations: 95% CI = 95% confidence intervals, *n* = unweighted sample size.

On the other hand, the overall prevalence of untreated coronal caries with regard to usual reason for visit was almost 1.8 times higher among problem‐oriented visitors (43.5%) than their counterparts (24.3%). The prevalence of untreated coronal caries increased from 43.7% at 15–34 years to 49.2% at 35–54 years and then declined to 30.9% at ≥ 75 years. Interestingly, 35–54‐year‐olds had the highest prevalence of untreated coronal caries for both problem‐oriented visitors (49.2%) and adults who usually visited for a check‐up (25.4%).

The mean number of decayed tooth surfaces per person (mean DS) in Australian adults according to age group and dental visiting patterns is displayed in Table 3. In general, the overall mean DS was 3 times greater in adults who made less frequent dental visits (2.1) than their more frequently visiting counterparts (0.7). This pattern was apparent across age groups, barring the oldest age group, with the mean DS increasing from 1.7 at 15–34 years to 3.3 at 55–74 years. Among the adults who usually made one or more dental visits per year, the lowest mean DS and the highest mean DS, respectively, were observed in 15–34‐year‐olds (0.6) and 35–54‐year‐olds (0.8). As for usual reason for visit, the overall mean DS was more than three times greater in problem‐oriented visitors (2.3) than their counterparts who visited for a check‐up (0.7), with a mean DS of problem‐oriented visitors and adults who visited for a check‐up, respectively, being the highest at 55–74 years (2.5) and 35–54 years (0.8). Interestingly, the mean DS was the lowest at ≥ 75 years, with the mean DS of problem‐oriented visitors and participants who visited for a check‐up being 1.8 and 0.5, respectively. There was no obvious variation in the mean DS with regard to use of a regular dentist overall or across age groups – the overall mean DS was 1.0 and 1.5, respectively, among people who did use a regular dentist and did not use a regular dentist.

**Table 3:** Mean number of decayed tooth surfaces per person in the Australian adults by age group and dental visiting patterns.

			Age (years)				
	*n*	% (95% CI)	Total Mean (95% CI)	15–34 Mean (95% CI)	35–54 Mean (95% CI)	55–74 Mean (95% CI)	≥ 75 Mean (95% CI)
Frequency of dental visits (%)							
≥ 1/year	2653	52.0 (49.2, 54.7)	0.70 (0.57, 0.84)	0.63 (0.39, 0.86)	0.81 (0.50, 1.12)	0.66 (0.49, 0.83)	0.70 (0.29, 1.10)
< 1/year	2254	48.0 (45.3, 50.8)	2.14 (1.77, 2.51)	1.65 (1.07, 2.22)	2.06 (1.56, 2.56)	3.27 (2.22, 4.33)	1.76 (0.69, 2.83)
Usual reason for visit (%)							
Check‐up	3135	61.5 (58.8, 64.1)	0.71 (0.57, 0.86)	0.68 (0.46, 0.90)	0.84 (0.50, 1.17)	0.64 (0.48, 0.80)	0.52 (0.31, 0.74)
Problem	1796	38.5 (35.9, 41.2)	2.28 (1.92, 2.65)	2.41 (1.53, 3.30)	2.14 (1.67, 2.60)	2.45 (1.73, 3.17)	1.82 (0.69, 2.94)
Use of a regular dentist (%)							
Yes	3444	73.6 (71.0, 76.1)	1.02 (0.83, 1.20)	1.18 (0.71, 1.65)	1.08 (0.73, 1.43)	0.85 (0.68, 1.01)	0.70 (0.35, 1.04)
No	967	26.4 (23.9, 29.0)	1.54 (1.13, 1.95)	1.26 (0.54, 1.99)	1.91 (1.30, 2.53)	1.60 (0.77, 2.43)	1.76 (0.38, 3.13)

Abbreviations: 95% CI = 95% confidence intervals, *n* = unweighted sample size.

Table 4 shows the mean number of decayed, missing, or filled tooth surfaces per person (mean DMFS) in Australian adults according to their age and dental visiting patterns. It was apparent that the mean DMFS increased across age groups in all adults notwithstanding their dental visiting patterns, with the youngest and the oldest adults in the respective dental visiting pattern categories reporting the lowest and the highest mean DMFS, respectively. With regard to the usual frequency of dental visits, the overall mean DMFS was 1.3 times higher in adults who usually made one or more dental visits per year (33.6) than adults who usually made less than one dental visits per year (25.7). However, across the age groups, this difference was obvious only at 55–74 years, with the mean DMFS being 60.6 for adults who made dental visits more frequently compared with that of their less frequently visiting counterparts (53.7). Analysis of DMFS by usual reason for visit revealed that the overall mean DMFS was nearly 1.3 times higher in problem‐oriented visitors (35.7) than their counterparts (26.6). This pattern was consistent only among 15–34‐year‐olds and 35–54‐year‐olds, with the mean DMFS successively increasing across these two age groups for both problem‐oriented visitors and participants who visited for a check‐up. On the other hand, the overall mean DMFS among adults who used a regular dentist was 1.5 times higher (34.4) than adults who did not use a regular dentist (22.6), although this pattern was not consistent across the age groups.

**Table 4:** Mean number of decayed, missing, or filled tooth surfaces per person in the Australian adults by age group and dental visiting patterns.

			Age (years)				
	*n*	% (95% CI)	Total Mean (95% CI)	15–34 Mean (95% CI)	35–54 Mean (95% CI)	55–74 Mean (95% CI)	≥ 75 Mean (95% CI)
Frequency of dental visits (%)							
≥ 1/year	2653	52.0 (49.2, 54.7)	33.58 (31.70, 35.46)	8.36 (7.28, 9.44)	26.44 (23.91, 28.97)	60.59 (57.67, 63.50)	78.08 (73.98, 82.17)
< 1/year	2254	48.0 (45.3, 50.8)	25.73 (24.02, 27.44)	6.91 (5.61, 8.20)	22.61 (20.49, 24.74)	53.72 (50.51, 56.93)	70.72 (66.00, 75.44)
Usual reason for visit (%)							
Check‐up	3135	61.5 (58.8, 64.1)	26.57 (24.99, 28.14)	6.48 (5.63, 7.34)	21.85 (19.80, 23.90)	56.74 (54.27, 59.20)	75.46 (71.11, 79.81)
Problem	1796	38.5 (35.9, 41.2)	35.66 (33.43, 37.90)	10.81 (8.93, 12.68)	29.83 (27.23, 32.44)	57.72 (53.50, 61.94)	75.29 (71.13, 79.45)
Use of a regular dentist (%)							
Yes	3444	73.6 (71.0, 76.1)	34.39 (32.73, 36.06)	8.65 (7.43, 9.86)	27.09 (24.81, 29.38)	59.00 (56.51, 61.48)	75.53 (71.97, 79.09)
No	967	26.4 (23.9, 29.0)	22.59 (19.96, 25.23)	7.73 (6.29, 9.17)	23.07 (19.55, 26.58)	55.86 (49.59, 62.13)	76.96 (71.40, 82.51)

Abbreviations: 95% CI = 95% confidence intervals, *n* = unweighted sample size.

### 3.2 Periodontal Diseases and Dental Visiting Patterns

The prevalence of gingivitis among Australian adults by age and dental visiting patterns is shown in Table 5. The overall prevalence of gingivitis was associated with the usual frequency of dental visits, with adults who usually visited less than once a year (33.5%) being 1.4 times more likely to have gingivitis than participants who visited once or more per year (23.9%). Across age groups, this pattern was obvious only at 55–74 years, with the lowest prevalence of gingivitis (18.5%) being observed in 55–74‐year‐olds who usually made one or more dental visits per year. The overall prevalence of gingivitis was 1.8 times higher among adults who usually visit for a problem (43.5%) than a check‐up (24.3%) and this pattern was consistent across age groups, up to 55–74‐year‐olds. Among all adults, the highest and lowest prevalence of gingivitis was reported by problem‐oriented visitors aged 35–54 years (49.2%) and adults who visited for a check‐up at ≥ 75years (19.5%), respectively. With regard to use of a regular dentist, people who did not use a regular dentist had 1.3 times higher prevalence of gingivitis (33.2%) compared with their counterparts who used a regular dentist (25.2%). However, there was no consistent variation in the prevalence of gingivitis with regard to use of a regular dentist, across the age groups.

**Table 5:** Proportion of Australian adults with gingival inflammation by age group and dental visiting patterns.

			Age (years)				
	*n*	% (95% CI)	Total% (95% CI)	15–34% (95% CI)	35–54% (95% CI)	55–74% (95% CI)	≥ 75% (95% CI)
Frequency of dental visits (%)							
≥ 1/year	2312	52.2 (49.3, 55.1)	23.9 (20.5, 27.6)	26.6 (21.1, 32.9)	25.1 (19.7, 31.3)	18.5 (14.1, 23.8)	21.4 (14.2, 31.0)
< 1/year	1985	47.8 (44.9, 50.7)	33.5 (29.8, 37.4)	33.3 (27.3, 40.0)	34.6 (28.8, 41.0)	33.8 (27.6, 40.6)	20.0 (12.0, 31.5)
Usual reason for visit (%)							
Check‐up	2775	62.3 (59.5, 65.1)	24.3 (21.4, 27.5)	24.2 (19.3, 29.9)	25.4 (20.3, 31.3)	24.4 (19.6, 30.0)	19.5 (13.1, 28.0)
Problem	1548	37.7 (34.9, 40.5)	43.5 (39.3, 47.9)	43.7 (35.6, 52.2)	49.2 (42.5, 56.0)	39.4 (32.5, 46.7)	30.9 (20.1, 44.3)
Use of a regular dentist (%)							
Yes	2979	73.2 (70.4, 75.8)	25.2 (22.0, 28.7)	27.5 (22.5, 33.1)	25.2 (20.2, 31.1)	20.7 (16.7, 25.3)	20.5 (13.6, 29.9)
No	880	26.8 (24.2, 29.6)	33.2 (29.3, 37.4)	35.4 (27.9, 43.6)	35.7 (29.3, 42.8)	28.7 (22.6, 35.8)	21.4 (12.2, 34.9)

Abbreviations: 95% CI = 95% confidence intervals, *n* = unweighted sample size.

Table 6 displays the prevalence of moderate or severe periodontitis among Australian adults according to their age and dental visiting patterns. In general, the moderate or severe periodontitis in Australian adults was age‐related, with its prevalence successively increasing across age groups of participants regardless of their dental visiting patterns, whereas the youngest and the oldest adults in the respective categories of dental visiting patterns reported the lowest and highest prevalence of moderate or severe periodontitis. The overall or age‐specific prevalence of moderate or severe periodontitis with regard to the usual frequency of dental visits did not show a consistent variation, with 29% and 30% adults who usually made one or more visits/year and less than one visit/year, respectively, experiencing moderate or severe periodontitis, on the whole. As for usual reason for visit, the overall prevalence of moderate or severe periodontitis was 1.4 times higher among problem‐oriented visitors (36.8%) than people who visited for a check‐up (26.1%). Across the age groups, this pattern was observed only at 15–34 years, with the youngest participants (aged 15–34 years) who visited for a check‐up having the lowest prevalence of moderate or severe periodontitis (8.8%). The overall prevalence of moderate or severe periodontitis was 31.7% and 25.6%, respectively, among adults who did and did not use a regular dentist. However, there was no obvious pattern in the prevalence of moderate or severe periodontitis with regard to use of a regular dentist in general or across the age groups.

**Table 6:** Proportion of Australian adults with moderate or severe periodontitis by age group and dental visiting patterns.

			Age (years)				
	*n*	% (95% CI)	Total % (95% CI)	15–34% (95% CI)	35–54% (95% CI)	55–74% (95% CI)	≥ 75% (95% CI)
Frequency of dental visits (%)							
≥ 1/year	2312	52.2 (49.3, 55.1)	29.0 (25.9, 32.4)	9.2 (6.2, 13.5)	31.0 (24.9, 37.9)	46.6 (40.4, 52.9)	72.7 (61.1, 81.9)
< 1/year	1985	47.8 (44.9, 50.7)	30.0 (26.5, 33.7)	14.6 (10.0, 20.7)	32.1 (26.0, 38.9)	55.8 (49.2, 62.2)	63.3 (49.4, 75.2)
Usual reason for visit (%)							
Check‐up	2775	62.3 (59.5, 65.1)	26.1 (23.4, 29.0)	8.8 (6.0, 12.9)	29.5 (23.9, 35.8)	49.0 (43.0, 55.0)	72.5 (60.4, 81.9)
Problem	1548	37.7 (34.9, 40.5)	36.8 (32.6, 41.3)	18.8 (13.4, 25.8)	37.2 (30.4, 44.5)	53.0 (45.6, 60.2)	64.3 (50.3, 76.2)
Use of a regular dentist (%)							
Yes	2979	73.2 (70.4, 75.8)	31.7 (28.9, 34.7)	10.2 (7.0, 14.6)	33.3 (28.0, 39.1)	48.7 (43.2, 54.2)	72.6 (63.1, 80.5)
No	880	26.8 (24.2, 29.6)	25.6 (20.6, 31.2)	14.3 (8.4, 23.4)	30.5 (21.5, 41.2)	57.1 (46.4, 67.2)	42.2 (21.3, 66.4)

Abbreviations: 95% CI = 95% confidence intervals, *n* = unweighted sample size.

## 4 Discussion

All three dental visiting patterns of dentate Australian adults that were assessed in the current study, namely, the usual frequency of dental visits, usual reason for dental visit, and the use of a regular dentist/dental practice were more likely to be affected by the year level of schooling and having dental insurance. For instance, higher proportions of adults from the categories of dentally uninsured and low year levels of schooling, respectively, were more likely to make dental visits less frequently and usually visit for a dental problem, whereas a higher proportion of adults with dental insurance used a regular dentist compared with their corresponding counterparts. In addition, residential location and the highest educational qualification attained were more likely to be associated with both frequency of dental visits and usual reason for visit, whereas eligibility for public dental care was more likely to be associated with usual reason for visit. For example, higher proportions of adults residing in major cities and participants with a degree or higher educational qualification, respectively, usually made dental visits more frequently and usually visited for a dental check‐up, whereas a higher proportion of adults who were eligible for public dental care usually visited for a dental problem, as opposed to their respective counterparts.

Overall, these findings point to socioeconomic gradients and social inequality in the provision of dental services and associated barriers to dental care in Australia. Dental insurance was the most conspicuously associated variable with all three dental visiting patterns under study. These findings reflect the delivery of dental care in Australia, which is predominantly driven by a fee‐for‐service private practice system, where dentally insured individuals have an opportunity to pay part or all their dental bill through their dental insurance, depending on their level of cover. Thus, people who have dental insurance are at an advantage of obtaining dental care, bypassing financial barriers associated with dental care, at least to a certain extent, as opposed to their dentally uninsured counterparts. It may also indicate fewer oral health issues and augmented oral health attitudes among dentally insured persons and persons with higher educational levels/qualifications, who usually visit for a dental check‐up rather that for a dental problem (Brennan et al. 2020; Teusner et al. 2013). On the other hand, relatively higher proportions of adults residing in rural/remote areas were found to be making less frequent dental visits as well as more visits for dental problems than their counterparts in major cities. This aligns with vast areas of geographic remoteness in Australia, which is represented by irregular distribution of dental practices alongside lower supply of dentists in remote areas across the country (Brennan et al. 2020; Dudko et al. 2018).

The present findings with regard to dental visiting patterns of Australian dentate adults and their dental caries experience revealed that the prevalence of dental caries was more likely to be associated with usual reason for visit. This was reflected by the presence of relatively higher levels of dental caries in problem‐oriented visitors than people who usually visited for a check‐up, overall and across the age groups, barring the oldest group. On the other hand, the severity and burden of untreated dental caries were higher among both problem‐oriented visitors as well as individuals who made dental visits less frequently, overall and across all but the oldest age group. Given that opportunities for regular dental treatment, including early diagnosis and appropriate treatment of asymptomatic dental decay, are less likely among not only those who visit their dental practitioner usually for a dental problem but also adults who make dental visits less frequently, there may be more likelihood of having a higher burden of untreated disease. We observed that the overall severity of dental caries experience or the lifetime experience of dental caries was associated with all three dental visiting patterns. The overall higher severity of dental caries experience among problem‐oriented visitors than their counterparts could be attributed to the reasons mentioned hitherto. Conversely, visiting a dental practitioner more frequently and using a regular dental practitioner may provide more opportunities for dental treatment as opposed to making dental visits less frequently and not using a regular dental practitioner. This was reflected in our findings, where adults who made dental visits more frequently and adults who used a regular dentist, respectively, were reported to experience more dental caries than their corresponding counterparts. Recommending dental visits every 6 months is still a common practice among dental practitioners worldwide. However, the available evidence with regard to this recommendation is weak and of low quality (Riley et al. 2013; Fee et al. 2020). On the one hand, individuals who make frequent dental visits are more likely to be provided with prompt preventive and therapeutic dental treatment; on the other hand, making such visits might potentially lead to iatrogenic overdiagnosis and overtreatment (Dudko et al. 2018; Al‐Asmar et al. 2022). Moreover, given that both the patient and the healthcare system have to bear the surging costs associated with overtreatment, there may be a prospect of making dental visits more frequently to be less cost‐effective (Dudko et al. 2018). Accordingly, our findings, which revealed a higher lifetime dental caries experience among Australian adults who made dental visits once or more per year compared with adults who made dental visits less frequently, may further cast doubt on the rationale behind recommending such fixed frequencies of dental visits, including biannual visits.

Analysis of dental visiting patterns of Australian adults by their periodontal disease experience revealed that the overall prevalence of gingivitis was higher among problem‐oriented visitors, adults who made dental visits less frequently, and individuals who did not use a regular dentist, whereas problem‐oriented visitors had consistently higher levels of gingivitis across the age groups, up to 55–74 years. On the other hand, the prevalence of moderate or severe periodontitis was more likely to be associated with usual reason for dental visit, where problem‐oriented visitors were more likely to have moderate or severe periodontitis than adults who visited for a check‐up. As such, the associations between dental visiting patterns and periodontal diseases were almost similar to those observed with regard to dental caries with problem‐oriented visitors specifically, and adults who made dental visits less frequently as well as did not have a regular dental practitioner in general, having higher prevalence of periodontal diseases. As discussed hitherto, such individuals may have less opportunities for regular periodontal treatment, including diagnosis and appropriate treatment of periodontal diseases at its early stage/s, which in turn lead them to have higher levels of gingivitis and moderate or severe periodontitis. Additionally, problem‐oriented visitors were more likely to have worse oral hygiene than their counterparts, who are usually visiting for a dental check‐up. This in turn can cause more plaque accumulation in their mouths and consequently, higher levels of gingivitis and periodontitis. Consistent with the previous studies (Kassebaum et al. 2014), our findings also supported the association of age with periodontal diseases—irrespective of dental visiting patterns, the prevalence of moderate or severe periodontal disease of Australian adults was consistently rising across the age groups.

Some of the key strengths of our study included using a nationally representative sample of Australian adults as well as using a standardized examination protocol and comprehensive epidemiological survey methods. In addition, due to the sufficient training that was provided to the interviewers and oral examiners, a high level of inter‐examiner reliability and agreement could be achieved, which in turn ensured quality control of the study. Determination of the causal associations was beyond the scope of our study, given its cross‐sectional design, whereas use of partial recording systems to record periodontal diseases might have underestimated the prevalence of periodontitis. Underrepresentation of Indigenous Australians was another limitation, which necessitated extra caution in interpreting the present findings with regard to Indigenous Australians.

## 5 Conclusions

Our findings revealed that Australian adults with low socioeconomic backgrounds, including adults who have had low levels of education as well as were residing in geographically remote locations, and in particular, individuals who did not possess dental insurance, were more likely to be problem‐oriented visitors, making dental visits less frequently, and not using a regular dentist. It was also observed that dental visiting patterns, including usually visiting for a dental problem, making dental visits less frequently, and not using a regular dentist, were more likely to be associated with higher levels of dental caries and periodontal diseases in Australian adults on the whole. In general, our findings support previous studies that have reported the existence of a socioeconomic gradient and the social inequality in the provision of dental services and associated barriers to dental care in Australia. It is envisaged that these findings may help clinicians to recognize patients at increased risk for dental caries and periodontal diseases while being useful for policy makers to plan and implement dental service provision, particularly among low socioeconomic groups, across states and territories in Australia.

## Author Contributions

Conceptualization: Najith Amarasena, Liana Luzzi, Sergio Chrisopoulos, and Gloria Mejia. Formal analysis: Sergio Chrisopoulos and Najith Amarasena. Writing – original draft preparation: Najith Amarasena. Writing – review and editing: Najith Amarasena, Liana Luzzi, Sergio Chrisopoulos, and Gloria Mejia. All authors have read and agreed to the published version of the manuscript.

## Ethics Statement

NSAOH was reviewed and approved by The University of Adelaide's Human Research Ethics Committee (HREC; H‐2016‐046).

## Consent

Interviewed subjects provided verbal consent before answering questions. Parental/guardian consent was obtained for participants aged 15–17 years. All examined subjects provided signed, informed consent before the examination (parents/guardians of participants aged 15–17 years provided signed, informed consent before the examination).

## Conflicts of Interest

The authors declare no conflicts of interest.

## Supporting information

Supporting information.

## Data Availability

The data sets used during the current study are available from the corresponding author via the completion of a data request.
