# Supplementary material for: Dental Visiting Patterns and Their Associations With Dental Caries and Periodontal Diseases
Source: Clin Exp Dent Res. 2025 Feb 12;11(1):e70087. doi: 10.1002/cre2.70087 (PMC11821545; doi:10.1002/cre2.70087)
Supplement: Supplementary file 1 — Supporting information. [file CRE2-11-e70087-s001.docx]

**Supplement file: Dental visiting patterns**

1. Frequency of dental visits ((≥1/year; <1/year):

This was assessed in the interview by asking dentate people ‘How often on average do you seek care from a dental professional?’ The responses included ‘Two or more times a year’, ‘Once a year’, ‘Once in two years’, ‘Less often than that’ or ‘Don’t know’. The first two response categories (‘Two or more times a year’ and ‘Once a year’) were combined into form the category of people who usually visit a dentist once or more/year ( ≥1/year) while others barring individuals who answered ‘Don’t know’ were included in <1 year category.

1. Usual reason for dental visit (check-up; problem):

Dentate Australian adults were asked ‘What is your usual reason for visiting a dental professional?’ Respondents are given the following options: ‘For a check-up’, ‘For a dental problem’, or ‘Don’t know’. Those who answered ‘Don’t know’ were excluded from the analysis while others were included in their corresponding category.

1. Use of a regular dentist/practice (yes; no):

Australian adults who were dentate and had made a dental visit within the last 5 years (people who had not made a dental visit in the last 5 years were considered as effectively out of dental care system and not included) were asked ‘Is there a dentist/practice you usually go to for dental care?’ People who answered ‘yes’ were regarded as using a regular dentist/practice.
